# The Efficacy and Safety of Different Dosages of Rituximab for Adults with Immune Thrombocytopenia: A Systematic Review and Meta-Analysis

**DOI:** 10.1155/2021/9992086

**Published:** 2021-10-06

**Authors:** Yu Dong, Ming Yue, Mengjiao Hu

**Affiliations:** ^1^Department of the Fourth Clinical Medical College, Zhejiang Chinese Medical University, Hangzhou, China; ^2^Department of Basic Medicine College, Zhejiang Chinese Medical University, Hangzhou, China

## Abstract

**Background:**

Rituximab has been frequently used as a second-line treatment for patients with immune thrombocytopenia (ITP). The optimal dose and course of rituximab are uncertain.

**Methods:**

A comprehensive search for randomized controlled trials reporting the use of low-dose (100 mg) or standard-dose (375 mg/m^2^) rituximab in ITP treatment was conducted. Meta-analyses were performed on CRR (complete response rate), ORR (overall response rate), PRR (partial response rate), SRR (sustained response rate), infection rate, SB (significant bleeding) rate, and SAE (serious adverse event) rate.

**Results:**

A total of 12 studies were included, comprising 869 patients. Compared to the control group, rituximab treatment resulted in an obvious increase in CRR (*P* < 0.00001), ORR (*P* < 0.0001), and SRR at month 6 and 12 (*P* = 0.0007, *P* = 0.0003), without increasing the infection rate (*P* = 0.12) and SAE rate (*P* = 0.11). No significant differences in CRR (RR 1.61 *vs.* 1.42, *P* = 0.45), ORR (RR 1.26 *vs.* 1.49, *P* = 0.28), PRR (RR 1.25 *vs.* 1.00, *P* = 0.11), SRR at month 12 (RR 2.00 *vs.* RR 1.64, *P* = 0.54), infection rate (RR 0.85 *vs.* 1.46, *P* = 0.36), and SB rate (RR 0.14 *vs.* 1.19, *P* = 0.17) were found in subgroups of low dose and standard dose.

**Conclusion:**

Rituximab was effective and safe for adult patients with ITP. A low-dose rituximab regimen might be an effective alternative to the standard-dose regimen in ITP, as it showed similar CRR, ORR, and SRR at month 12 and was relatively safer with a lower cost.

## 1. Introduction

Immune thrombocytopenia (ITP) is an autoimmune disorder characterized by a platelet count <100 × 10^9^/L, in the absence of known conditions that could be associated with thrombocytopenia [1]. The main mechanisms leading to thrombocytopenia in these patients are increased peripheral immune-mediated platelet destruction and impaired platelet production by megakaryocytes [2]. Corticosteroid treatment is the standard first-line therapy, with which 60%-80% of patients achieve an initial response [3, 4]. However, relapse is common, and only 30%-50% of patients achieve a durable response after the discontinued treatment of corticosteroids. Patients who failed to have an initial response to corticosteroids or relapsed are recommended to receive second-line treatment [5]. Several second-line treatments, including immunosuppressive agents (*i.e.*, azathioprine), monoclonal antibodies (*i.e.*, rituximab), thrombopoietin receptor agonists (TPO-RAs, *i.e.*, eltrombopag), and splenectomy, have been used. Splenectomy usually leads to sustained remission in up to 70% of patients [6]. Nonetheless, patients may not incline to choose splenectomy as it is an irreversible surgery [7]. TPO-RA treatment has a high remission rate of 80%. However, it does not address the root cause of platelet destruction and requires long-term standardized medication, which places a heavy economic burden on patients. Moreover, the disease is liable to relapse when the treatment is interrupted [8].

B cell depleting therapies such as rituximab have been widely used in the ITP second-line treatment due to the significant role of B cells in the pathogenesis of ITP [2]. The anti-CD20 chimeric monoclonal antibody rituximab binds to CD20 and triggers B cell depletion by various mechanisms, such as apoptosis, complement-dependent cytotoxicity, and antibody-dependent cell-mediated cytotoxicity [9]. The complete depletion of B cells in the blood, spleen, and bone marrow is achieved within the first several weeks after rituximab infusion. Response rates of 40% and 30% at 1 and 2 years of follow-up are usually obtained [10], and 21%-26% of patients are still responders after five years [11], which indicates the rituximab remission could last for an extended period. According to the recommendations of ASH (American Society of Hematology) and International Working Group (IWG) consensus [12, 13], a standard dose regimen of rituximab is 375 mg/m^2^ weekly for 4 weeks. This standard dosage regimen demonstrates the effectiveness of a 69% overall initial response rate and a 35% sustained response rate [14].

With the purpose of minimizing the incidence of adverse events and reducing the cost of treatment, some studies have begun to apply low-dose rituximab (100 mg or 100 mg/m^2^ weekly for 4 weeks) in the treatment of ITP. This low-dose regimen shows a 60.5% overall initial response rate, which is similar to the standard regimen [15]. At present, both dosages of rituximab are applied in clinical treatment, while which is better remains controversial since there lack of comparisons between these two dosages. Therefore, in order to provide a basis for rational clinical medication, we reported a meta-analysis designed systematically to evaluate the efficacy and safety of different dosages of rituximab in adult patients with ITP.

## 2. Materials and Methods

This meta-analysis was conducted in accordance with PRISMA (Preferred Reporting Items for Systematic Reviews and Meta-Analysis) Statement [16]. The study protocol was registered in the PROSPERO database (CRD42020190856).

### 2.1. Search Strategy and Eligibility Criteria

We systematically searched MEDLINE (PubMed), EMBASE, the Cochrane Library, ClinicalTrials.gov, China National Knowledge Infrastructure (CNKI), Wanfang, and Weipu (VIP) databases from January 1990 to June 8, 2020. There were no language restrictions to the search. We conducted a literature search using controlled vocabularies, such as Medical Subject Headings (MeSH) or Emtree, and free text words, including “purpura, thrombocytopenic, idiopathic”, “immune thrombocytopenia”, “autoimmune thrombocytopenia”, “immune thrombocytopenic purpura”, “autoimmune thrombocytopenic purpura”, “rituximab”, “rituxan”, “GP2013”, “anti-CD20”, “IDEC-C2B8” or “mabthera”. The exact search queries were modified for each database. The complete search strategies were presented in the supplemental data.

Studies were included if they met the following criteria:

(1) Study type: randomized controlled trial
(2) Patient: any race, aged ≥18 years and diagnosed with immune thrombocytopenia
(3) Intervention: use of rituximab in any dosage, with or without combination therapy
(4) Comparison: nonrituximab treatment or placebo
(5) Outcome: the following indicators which were reported from the studies:
  - Complete response (CR): defined as a platelet count ≥100 × 10^9^/L measured on two occasions more than 7 days apart, and the absence of bleeding
  - Overall response (OR): defined as a platelet count ≥50 × 10^9^/L more than 7 days apart, and the absence of bleeding
  - Partial response (PR): defined as a platelet count ≥30 × 10^9^/L or a greater than 2-fold increase in platelet count from baseline more than 7 days apart, and the absence of bleeding
  - Sustained response (SR): as defined in primary studies
  - Infection
  - SB (Significant bleeding): as defined in the primary studies
  - SAEs (serious adverse events): as defined in the primary studies

Studies were excluded if they met the following criteria: (1) patients diagnosed with secondary immune thrombocytopenia, (2) trials without extractible data, and (3) duplicate publications.

### 2.2. Data Extraction and Quality Assessment

Two investigators (Y.D. and M.H.) independently screened titles and abstracts of the studies retrieved according to predefined eligibility criteria. Potentially related studies were further judged based on full-text screening and inclusion criteria. Data were then extracted from the included studies by two independent investigators (Y.D. and M.H.), including (1) study characteristics (authors, publication year, the country where the study was conducted, funding sources, study ID, study design, and participant demographics); (2) baseline characteristics (age, ITP stage of patients, gender, follow-up time, and platelet count before treatment); and (3) outcome events (number of patients who achieved CR, OR, PR, SR, number of patients who experienced infection, significant bleeding, and serious adverse event). Discrepancies about study selection and data extraction were resolved by discussion.

The quality of the included studies was assessed by two independent reviewers (Y.D. and M.H.) using the Cochrane risk of the bias assessment instrument. The following sources of bias were evaluated: random sequence generation, allocation concealment, blinding of participants and personnel, blinding of outcome assessment, incomplete outcome data, selective reporting, and other bias. Each item was graded as “low risk” or “high risk”; if there was insufficient information to judge, it was classified as “unclear”. Disagreements about quality assessment were resolved by discussion.

### 2.3. Statistical Analysis

All outcomes were dichotomous data calculated using risk ratio (RR) with the corresponding 95% confidence interval (CI). The level of statistical heterogeneity was defined by using *I*^2^ test. A fixed-effect model (Mantel-Haenszel method) was used if *I*^2^ < 50%; otherwise, a random-effect model approach was adopted. Sensitivity analysis was conducted to test the possible influence of every study and explore the robustness of the results by eliminating possible extreme observations. All statistical analyses were conducted using RevMan version 5.3 (Nordic Cochrane Center, Copenhagen, Denmark).

## 3. Results

### 3.1. Study Selection

**Figure 1:**
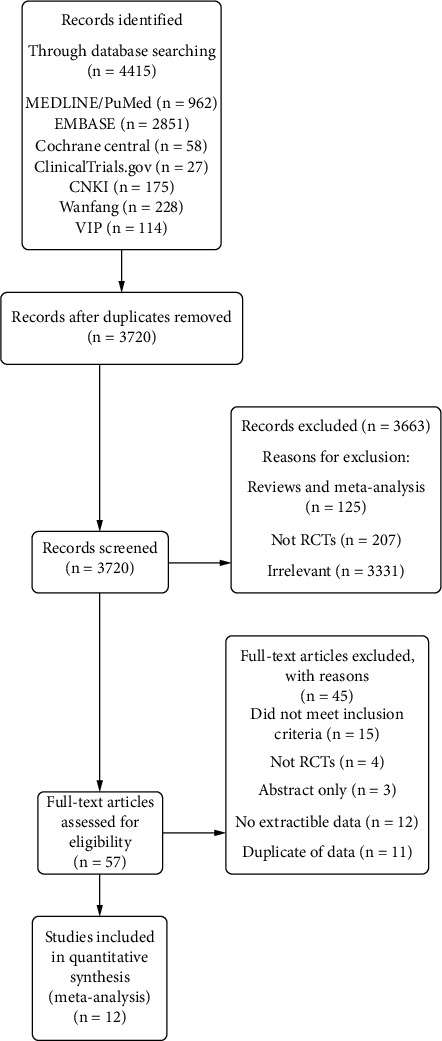
PRISMA flow diagram of the screening and selection process used in the study.

A total of 4415 records were identified through the initial search. After removing duplicates, 3720 studies were screened by titles and abstracts, and 57 records were left for full-text review. Finally, 12 studies [17–28] that met the eligibility criteria were included in the meta-analysis (Figure 1).

### 3.2. Study Characteristics

The baseline characteristics of these 12 studies were described in Table 1. All studies were randomized controlled trials, with sample sizes ranging from 46 to 133. In total, 869 patients were analyzed; among them, 494 were females, and the percentage of females ranged from 29% to 73% among those studies. Studies were published between 2010 and 2019, five of which were written in English, and seven were written in Chinese.

Rituximab was intravenously administered at the standard dose (375 mg/m^2^) weekly for 4 weeks in 5 studies and at low dose (100 mg) weekly for 4 weeks in 7 studies. Two trials compared rituximab treatment with placebo. Eight trials compared rituximab plus dexamethasone versus dexamethasone monotherapy. One trial compared rituximab combined with dexamethasone and taper-dose prednisone to dexamethasone and taper-dose prednisone. One trial compared rituximab combined with cyclophosphamide versus cyclophosphamide monotherapy.

### 3.3. Quality Assessment

**Figure 2:**
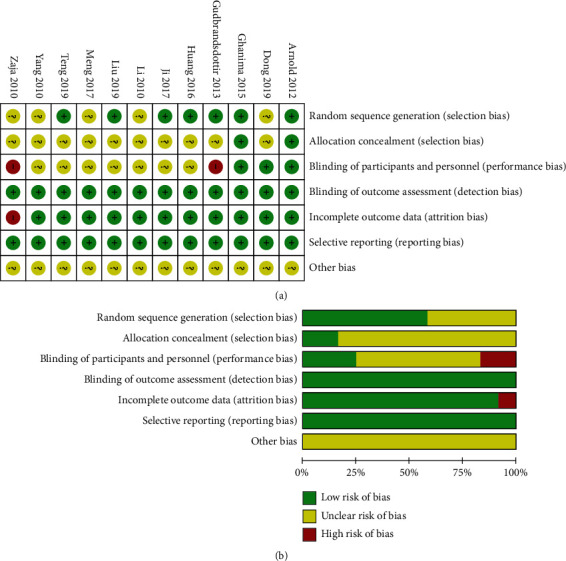
Summary (a) and graph (b) of the risk of bias in the included trials by the Cochrane risk of bias assessment instrument. Assessments were based on the reviewers' judgment of each domain.

Two studies [19, 21] were not double-blind and therefore considered high risks of performance bias. One study [21] was regarded as high risk in attrition bias as more than 50% of patients discontinued the study. Four RCTs [17–19, 21] were sponsored by Roche, which produced the drugs used in the trials. Overall, all the included studies had a low risk of bias as evaluated by the Cochrane risk of bias assessment instrument. The result was presented in Figure 2.

### 3.4. Efficacy Analysis

#### 3.4.1. Complete Response Rate

**Figure 3:**
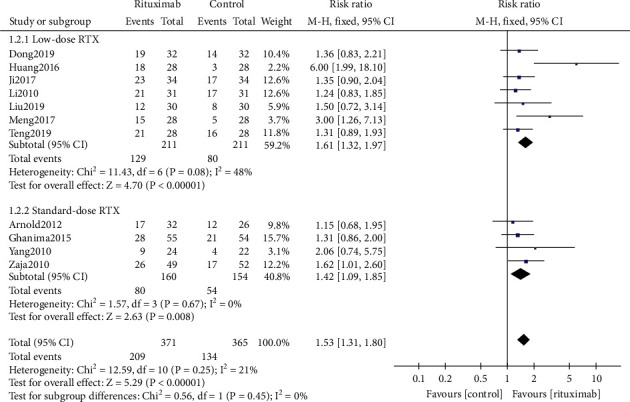
Forest plots of risk ratio in CRR. RTX: rituximab. CI: confidence interval; M-H: Mantel-Haenszel.

The complete response rate (CRR) was conducted from 11 trials [17, 18, 20–28] (*n* = 736). Compared to the patients who received nonrituximab treatment or placebo, patients who received rituximab treatment were more likely to achieve a complete response (RR 1.53, 95% CI (1.31, 1.80), *P* < 0.00001, Figure 3). Both the low-dose rituximab subgroup and the standard-dose rituximab subgroup had shown great efficiency on CRR (RR 1.61, 95% CI (1.32, 1.97), *P* < 0.00001*vs.* RR 1.42, 95% CI (1.09, 1.85), *P* = 0.008, Figure 3), while there was no significant difference between the subgroups (*P* = 0.45). There was moderate heterogeneity (*I*^2^ = 48%, *P* = 0.08) in the CRR indicator of the low-dose rituximab subgroup, which may be caused by the better effect size of the study of Huang et al. compared to other trials. With the removal of the study, heterogeneity disappeared (RR 1.44, 95% CI (1.18, 1.76), *P* = 0.0004; heterogeneity *I*^2^ = 0%, *P* = 0.59, Supplement figure 1). The meta-analysis result was not reversed after removing the study, which indicated the robustness of the result.

#### 3.4.2. Overall Response Rate

**Figure 4:**
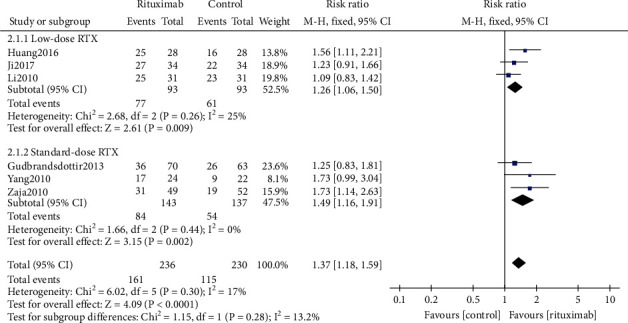
Forest plots of risk ratio in ORR. RTX: rituximab. CI: confidence interval; M-H: Mantel-Haenszel.

A total of 466 patients from 6 studies [19–21, 23, 24, 28] were assessed for the treatment's overall response rate (ORR). Pooled analysis by using the fixed-effect model revealed significantly higher efficiency of ORR in the rituximab group than in the control group (RR 1.37, 95% CI (1.18, 1.59), *P* < 0.0001, Figure 4). No statistically significant difference was found between the subgroups of the low-dose and the standard-dose rituximab (RR 1.26, 95% CI (1.06, 1.50), *P* = 0.009*vs.* RR 1.49, 95% CI (1.16, 1.91), *P* = 0.002; test for subgroup differences *P* = 0.28, Figure 4).

#### 3.4.3. Partial Response Rate

**Figure 5:**
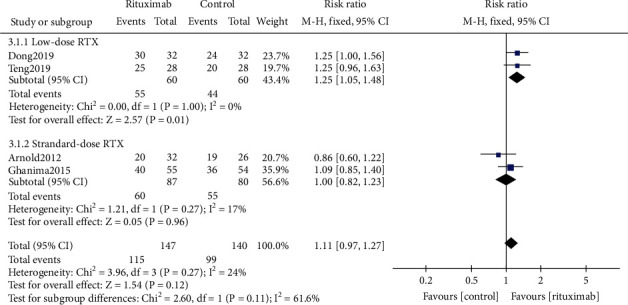
Forest plots of risk ratio in PRR. RTX: rituximab. CI: confidence interval; M-H: Mantel-Haenszel.

Four studies [17, 18, 22, 27] reported partial response rate (PRR) (*n* = 287). In contrast with the control group, the rituximab group did not show a more effective partial response (RR 1.11, 95% CI (0.97, 1.27), *P* = 0.12, Figure 5). The low-dosage rituximab treatment was found associated with a better PRR than the standard dosage treatment (RR 1.25, 95% CI (1.05, 1.48), *P* = 0.01*vs.* RR 1.00, 95% CI (0.82, 1.23), *P* = 0.96, Figure 5), while the difference between the subgroups was not statistical (*P* = 0.11).

#### 3.4.4. Sustained Response Rate

**Figure 6:**
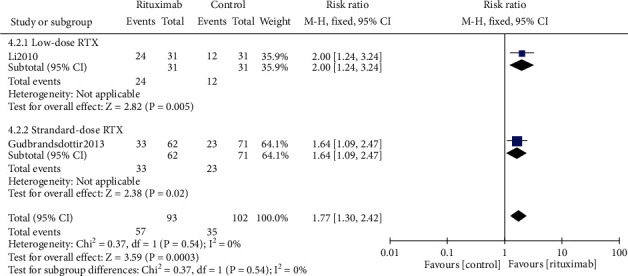
Forest plots of risk ratio in SRR at month 12. RTX: rituximab. CI: confidence interval; M-H: Mantel-Haenszel.

Three trials [19–21] reported sustained response rate (SRR), two [19, 21] of which reported SRR at month 6 and two [19, 20] of which reported SRR at month 12. Two analyses were performed by a fixed-effect model, which manifested that patients who received rituximab were more likely to achieve a sustained response at month 6 and 12 (RR 1.61, 95% CI (1.22, 2.12), *P* = 0.0007; RR 1.77, 95% CI (1.30, 2.42), *P* = 0.0003, Supplement figure 2 and Figure 6). Both the low-dose rituximab subgroup and the standard-dose rituximab subgroup showed great efficiencies on SRR at month 12 (RR 2.00, 95% CI (1.24, 3.24), *P* = 0.005*vs*. RR 1.64, 95% CI [(1.09, 2.47), *P* = 0.02, Figure 6), while there was no statistical difference between the subgroups (*P* = 0.54).

### 3.5. Safety Analysis

#### 3.5.1. Infection Rate

**Figure 7:**
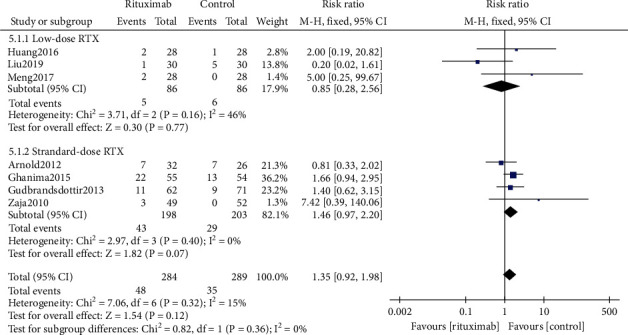
Forest plots of risk ratio in infection. RTX: rituximab. CI: confidence interval; M-H: Mantel-Haenszel.

Seven trials [17–19, 21, 23, 25, 26] (*n* = 573) reported infection events, which happened to 48 of 284 (16.9%) patients in the rituximab group compared to 35 of 289 (12.1%) patients in the control group. There was no significant difference between the rituximab group and the control group in the incidence of infection (RR 1.35, 95% CI (0.92, 1.98), *P* = 0.12, Figure 7). Subgroup analysis indicated that the incidence of infection in the low-dose rituximab subgroup was lower than that in the standard-dose subgroup (RR 0.85, 95% CI (0.28, 2.56), *P* = 0.77*vs.* RR 1.46, 95% CI (0.97, 2.20), *P* = 0.07, Figure 7), but the difference between subgroups was not statistically significant (*P* = 0.36). The common infections reported were upper respiratory tract infections, pneumonia, influenza, topical infections, pyrexia, and bronchitis.

#### 3.5.2. Significant Bleeding Rate

Details of significant bleeding events were reported by five studies [17–19, 21, 23], which used the Page immune thrombocytopenia bleeding score [17], the Clinical Terminology Criteria for Adverse Events (CTCAE) instrument [19, 21], and the Khellaf bleeding score [18] to evaluate the bleeding events. SB was defined as grade 2 or higher (Page score) [29], grade 3 or higher (CTCAE definition), or a weighted cumulative score above 8 (Khellaf score) [30].

**Figure 8:**
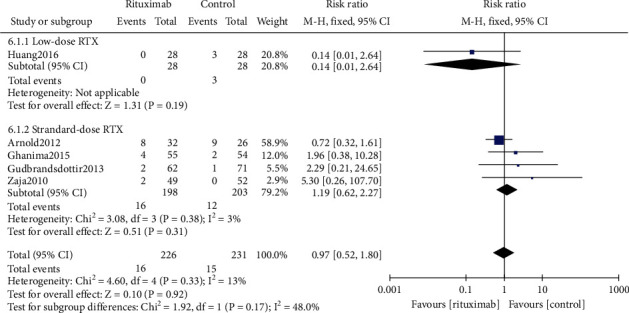
Forest plots of risk ratio in SB. RTX: rituximab. CI: confidence interval; M-H: Mantel-Haenszel.

There was no statistical difference between the rituximab and control groups in the SB indicator (RR 0.97, 95% CI (0.52, 1.80), *P* = 0.92, Figure 8). For the standard-dose rituximab subgroup and the low-dose rituximab subgroup, when compared to their control groups in the SB indicator, respectively, there was no statistical difference neither (RR 1.19, 95% CI (0.62, 2.27), *P* = 0.61*vs.* RR 0.14, 95% CI (0.01, 2.64), *P* = 0.19, Figure 8). Besides, there was no statistical difference between the subgroups in SB rate (*P* = 0.17).

#### 3.5.3. Serious Adverse Events (SAEs) Rate

Three trials [17, 18, 21] of the standard-dose rituximab subgroup reported SAEs. The incidence of SAEs in the rituximab group did not differ from the control group (RR 1.74, 95% CI (0.89, 3.40), *P* = 0.11, Supplement Figure 3).

## 4. Discussion

Rituximab has been a widely used second-line therapy in ITP. The optimal dose regimen of rituximab in ITP treatment remains controversial. As far as we are aware, this is the first systematic review and meta-analysis of randomized controlled trials to address this question.

The efficacy of treatment is a significant part of our meta-analysis. Our review observed that rituximab could significantly increase the CRR and the ORR after treatment compared to the control group. Moreover, no significant differences in primary efficacy outcomes, including the CRR (*P* = 0.45), the ORR (*P* = 0.28), and the PRR (*P* = 0.11), were found between the standard-dose rituximab subgroup and the low-dose one. These results suggested that the short-term efficacies of the two dosage regimens were similar. In a previous meta-analysis of observational and randomized trials, the standard-dose rituximab treatment showed CR in 41% of patients and OR in 57% of patients [31]. In another meta-analysis of observation trials, patients were treated with the standard-dose rituximab, 43.6% of patients achieved CR, and 62.5% of patients achieved OR [32]. Furthermore, a meta-analysis of observation trials reported the low-dose rituximab was associated with a CRR of 44% and an ORR of 63% [33]. According to the data from our meta-analysis and the meta-analysis of observational studies, the conclusion is that in ITP treatment, the low-dose rituximab and the standard-dose rituximab have similar short-term clinical efficacy.

When evaluating the duration of response (DoR) of rituximab, our meta-analysis indicated rituximab could significantly increase the SRR, and the SRR at month 12 was comparable between the low-dose rituximab subgroup and the standard-dose subgroup (*P* = 0.54). A retrospective review compared the efficacy of low- and high-dose rituximab in ITP patients; SRR at month 6 was 37.7% and 43.1% in the low- and standard-dose regimen, respectively [34]. In a long-term follow-up trial, it was reported that the duration of response of low-dose rituximab and standard-dose rituximab were 22 (range from 3–52) months and 21 (3–120) months, respectively (*P* = 0.148). After CR, the relapse rate in the low-dose rituximab group was 14%, significantly lower than the standard-dose group (37.5%). However, the long-term response rates were 24% and 41% with the relapse rates of 54% *vs.* 38%, and the estimated 4-year projected EFS (event-free survival) was 23% and 35%, in the low-dose and the standard-dose rituximab group, respectively (*P* = 0.1228) [35]. Another contrary report mentioned that data from 17 published studies, including 376 adults, showed a five-year response rate of 21% to the standard dose rituximab [11]. On these grounds, loss of response was more probable when the interval between diagnosis and rituximab therapy was longer. These data indicated that the duration of response in the low-dose and standard-dose rituximab subgroups were comparable at least in one year. In the future, with more clinical trials, we can more precisely assess the long-term response rate of the low-dose rituximab regimen.

The safety of drugs is an essential consideration in clinical practice, especially for chronic diseases like ITP. The significant bleeding and infection were regarded as primary indicators of the safety evaluation because ITP patients owned increased risks of bleeding, and long-term treatment of rituximab led to the B-cell depletion resulting in immunosuppressive effect in ITP patients. Our review revealed that rituximab was not associated with a reduction in significant bleeding or an increase in infection and SAEs. There was no significant difference in the incidence of significant bleeding (*P* = 0.17) and infection (*P* = 0.36) between the low-dose and standard-dose rituximab subgroups. What is more, the low- dosage rituximab treatment had a lower infection rate than the standard dosage treatment (RR = 0.85*vs*. *RR* = 1.46). However, it is worth noting that the short observation periods of randomized controlled trials may lead to the infection events not being fully recorded.

Infusion reaction was a joint adverse event of rituximab. Health authorities have recommended 100 mg methylprednisolone for systemic premedication to prevent the infusion reaction [36]. We could not conduct a formal statistical analysis of such events as only one trial has reported infusion reaction events in the control group [17]. In fact, most of the patients in the included randomized controlled trials had systemically received premedication to prevent infusion reactions, which may explain why restricted infusion reactions were reported. Moreover, according to a meta-analysis of observational studies, the incidence of infusion reactions in patients treated with the standard dosage of rituximab is approximately 18% [32]. In another meta-analysis, 17.5% of patients treated with the low dosage of rituximab were reported to experience infusion reactions [33]. These observed infusion reactions were well tolerated, no severe or fatal event was reported. With the consecutively weekly injection, the response would gradually be weakened or disappeared without affecting the treatment. Among the included studies, most of the adverse events related to the rituximab were mild and moderate (grade1-2). Only three trials reported severe adverse events, but no statistical differences were shown between the intervention and control groups. These findings suggest rituximab is relatively safe for patients with ITP, and the low-dosage regimen may have a better safety profile than the standard-dosage regimen.

The cost of care for adult patients with chronic ITP is expensive, especially for patients with severe disease conditions. It should be noted that the cost of the treatment remains a critical factor in determining the clinical treatment plan. Each patient treated with the standard rituximab dosage should receive a total dose of about 2800 mg [37]. The cost of the standard dose regimen of rituximab in treating ITP is about 10000-40000 USD per 4-infusion course [38]. The high price of rituximab limits the clinical application of the medicine, especially for patients with poor economic conditions. In contrast, patients treated with the low-dosage regimen only have to receive a total dose of 400 mg of rituximab, which may significantly reduce the medical burden of ITP patients.

Based on the efficacy, safety, and cost-effectiveness, we consider that low-dose rituximab has good clinical application value. However, it is still challenging to determine which clinical period patients are suitable for low-dose rituximab and with which combination therapy is the optimal setting for low-dose rituximab. The ASH guideline panel recommends rituximab to patients with a disease duration of fewer than 12 months and who prefer avoiding surgery or long-term medication [39], and earlier administration of rituximab might lead to a higher long-term response rate [40]. Moreover, according to our literature review, in ITP treatment, rituximab plus combination therapy yielded a higher response rate than rituximab monotherapy (Supplement table 1). Besides, the International Working Group (IWG) has recommend the administration of dexamethasone at 40 mg/day for 4 days as the first-line treatment in ITP [1]. Therefore, we consider that 100 mg rituximab weekly for 4 weeks plus 40 mg dexamethasone daily for days 1-4 might be the optimal choice for a low-dose regimen.

Except for the standard-dose and low-dose regimens, there were two uncommonly used dose schemes: one was a double-standard dose regimen (750 mg/m^2^ weekly for 4 weeks), and the other one was the 1000 mg rituximab (on day 1 and 15), which was firstly used in rheumatoid arthritis treatment. Compared with the standard-dosage regimen, the double-standard dose regimen neither increased the response rate nor proved superior to the standard dosage regimen [41]. The 1000 mg dosage regimen of rituximab was reported by a retrospective study and a prospective study, which seemed to achieve similar efficacy to the standard dose regimen, and might be used as an alternative to the standard dose one [42, 43]. More studies, especially the randomized controlled trials, were needed to verify these findings.

Throughout the years, several factors of patient characteristics have been investigated to predict response to rituximab. Some studies have pointed out that young age, female sex, achievement of complete response, and short disease duration might be related to a durable rituximab response [44–46]. However, some contradictory results have reported that young age and gender female were not predictive factors [11, 36]. Actually, up to now, there is no credible patient characterized factor for predicting the efficacy of rituximab. The possible mechanism of rituximab for ITP treatment is preplasma B cells could be depleted by rituximab, which leads to the reduction of antiplatelet autoantibodies (APA). Several studies have investigated the correlation between APA levels and the response to rituximab. Cooper et al. pointed out that the decreased APA levels were associated with the increased platelet count, and they occurred concurrently [47]. The findings of Porcelijn et al. showed that the response to rituximab appeared strongly associated with a reduction in platelet-bound antibodies, which suggested the correlation between the absence of platelet-bound antibodies and the refractoriness to rituximab [48]. However, Arnold et al. proposed that neither APA's existence at baseline nor the vanishment of APA after treatment was related to a response to rituximab. Despite that, the persistent autoantibodies after the treatment can be a marker of disease severity [49]. These conflicting results suggested that the biological predictors of the response to rituximab remain to be investigated further.

There were several discrepancies in study designs, ITP stage of patients, the treatments before rituximab, and combined therapy in the included trials. This meta-analysis was not a direct comparison between the low-dose and standard-dose rituximab in the treatment of ITP. However, owing to the fact that the combination therapy of the intervention group and the control group was the same and rituximab was the only variable, we can still compare them indirectly by using subgroup difference of RR values. We conducted a subgroup and sensitivity analysis to test the difference between the rituximab and rituximab plus combination therapy. The results revealed that in low-dose rituximab treatment, “100 mg RTX plus 2 mg/kg CTX “ *vs.* “2 mg/kg CTX” subgroup showed CRR differences (*P* = 0.02, Supplement table 2). Beyond that, no subgroup differences were found between the rituximab subgroup and the rituximab plus combination therapy subgroup. These data further confirmed that the not unified combination therapy in the low-dose rituximab subgroup or the standard-dose rituximab subgroup would not affect the data combination (Supplement table 2 and table 3). Besides, the sample size of included studies was small. The short follow-up period ranging from 4 weeks to 19.5 months might lead to the safety events not being fully recorded. Moreover, the different approaches of reporting and multiple measurement tools used may induce the bias of bleeding assessment [50].

## 5. Conclusion

In summary, this systematic review and meta-analysis results indicated that the low-dose rituximab regimen might be an effective alternative to the standard-dosage regimen, especially in a resource-limited setting, as it showed similar short-term efficacy and response duration and was relatively safer with a lower cost. The longtime follow-up head-to-head trial of the low-dosage regimen and the standard-dosage regimen will ultimately provide more comprehensive and detailed information for physicians and patients.

## Figures and Tables

**Table 1:** Baseline characteristics of included studies.

Study	Patients, *n*	Females, *n* (%)		Age (years), mean (SD) or median (range)`		Intervention	Comparison	Outcomes	Follow-up
		EXP	CTRL	EXP	CTRL				
*Standard-dose RTX subgroup*									
Zaja et al. [21]	101	27 (55)	33 (63)	49.0 (16.0)	47.0 (19.0)	RTX: 375 mg/m^2^, weekly, 28 d DXM: 40 mg, qd, 1-4d	DXM: 40 mg, qd, 1-4 d	CR, OR, SR, SB, SAEs, infection	6 months
Gudbrandsdottir et al. [19]	133	36 (58)	34 (48)	51 (36-63)	58 (41-70)	RTX: 375 mg/m^2^, weekly, 28 d DXM: 40 mg, qd, 1-4d	DXM: 40 mg, qd, 1-4 d	OR, SR, SB, SAEs, infection	6 months
Yang et al. [28]	46	14 (58)	13 (59)	47 (26-68)	50 (28-69)	RTX: 375 mg/m^2^, weekly, 28 d DXM: 10 mg, qd, 1-4 d	DXM: 40 mg, qd, 1-4 d	CR, OR	3 months
Ghanima et al. [18]	109	40 (73)	39 (72)	46 (27-61)	46 (28-60)	RTX: 375 mg/m^2^, weekly, 28 d	Placebo	CR, PR, SB, infection	19.5 months
Arnold et al. [17]	58	19 (58)	16 (59)	40 (30-59)	40 (31-59)	RTX: 375 mg/m^2^, weekly, 28 d	Placebo	CR, PR, SB, SAEs, infection	6 months
*Low-dose RTX subgroup*									
Li et al. [20]	62	18 (58)	19 (61)	26 (18-51)	24 (18-59)	RTX: 100 mg, weekly, 28 d DXM: 40 mg, qd, 1-4 d PRE: taper, 28 d	DXM: 40 mg, qd, 1-4 d PRE: taper, 28d	CR, OR, SR	12 months
Meng et al. [26]	56	10 (36)	8 (29)	38 (18-65)	35 (18-66)	RTX: 100 mg, weekly, 28 d DXM: 40 mg, qd, 1-4 d	DXM: 40 mg, qd, 1-4 d	CR, infection	4 weeks
Ji et al. [24]	68	16 (47)	14 (41)	32.4 (2.5)	33.0 (2.2)	RTX: 100 mg, weekly, 28 d DXM: 40 mg, qd, 1-4 d	DXM: 40 mg, qd, 1-4 d	CR, OR	6 months
Dong et al. [22]	64	21 (66)	20 (63)	35.5 (5.0)	35.5 (4.6)	RTX: 100 mg, weekly, 28 d DXM: 40 mg, qd, 1-4 d	DXM: 40 mg, qd, 1-4 d	CR, PR	4 weeks
Liu et al. [25]	60	14 (47)	13 (43)	42.7 (1.6)	42.5 (1.2)	RTX: 100 mg, weekly, 28d DXM: 3 mg, tid, 1-4d	DXM: 3 mg, tid, 1-4 d	CR, infection	4 weeks
Teng et al. [27]	56	18 (64)	17 (61)	37.0 (1.6)	36.6 (2.2)	RTX: 100 mg, weekly, 28 d DXM:1 mg/kg, qd, 1-4 d	DXM:1 mg/kg, qd, 1-4 d	CR, PR	4 weeks
Huang et al. [23]	56	16 (57)	19 (68)	33.0 (4.0)	34.0 (4.0)	RTX: 100 mg, weekly, 28 d CTX: 2 mg/kg, qd, 2-3 m	CTX: 2 mg/kg, qd, 2-3 m	CR, OR, SB, infection	6 months

EXP: experience group; CTRL: control group; RTX: rituximab; DXM: dexamethasone; PRE: prednisone; CTX: cyclophosphamide; qd: once a day; tid: three times a day; CR: complete response (platelet count ≥ 100 × 10^9^/L); OR: overall response (platelet count ≥ 50 × 10^9^/L); PR: partial response (platelet count ≥ 30 × 10^9^/L); SB: significant bleeding; SAEs: serious adverse events.

## Data Availability

Previously reported data of clinical trials was used to support this study and are available at the following: [doi:10.1182/blood-2011-08-374777; doi:10.1016/S0140-6736(14)61495-1; doi:10.1182/blood-2012-09-455691; doi:10.1007/s12185-010-0753-z; doi:10.1182/blood-2009-07-229815; https://kns.cnki.net/kcms/detail/detail.aspx?dbcode=CJFD&dbname=CJFDLAST2019&filename=XTYX201915001; https://kns.cnki.net/kcms/detail/detail.aspx?dbcode=CJFD&dbname=CJFDLAST2016&filename=YXZS201603048; https://kns.cnki.net/kcms/detail/detail.aspx?dbcode=CJFD&dbname=CJFDLAST2017&filename=WMIA201702104; https://kns.cnki.net/kcms/detail/detail.aspx?dbcode=CJFD&dbname=CJFDLAST2019&filename=ZGUD201930019; https://kns.cnki.net/kcms/detail/detail.aspx?dbcode=CJFD&dbname=CJFDLAST2017&filename=EBED201702010; https://kns.cnki.net/kcms/detail/detail.aspx?dbcode=CJFD&dbname=CJFDLAST2019&filename=SXYZ201901032; https://kns.cnki.net/kcms/detail/detail.aspx?dbcode=CJFD&dbname=CJFD2010&filename=HKHT201011052]. These prior studies (and datasets) are cited at relevant places within the text as references [17–28].

## Additional Points

*Highlights*. Rituximab is an effective and safe medication for adults with immune thrombocytopenia. The low-dose rituximab regimen has similar efficacy in immune thrombocytopenia compared with the standard-dose regimen. The low-dose rituximab regimen has better safety profile than the standard-dose regimen. The low-dose rituximab regimen can reduce the medical burden of patients with immune thrombocytopenia than the standard-dose regimen.

## Supplementary Materials

Supplementary Materials Supplement Figure 1: forest plots of risk ratio in CRR after removing the research of Huang et al. RTX: rituximab. CI: confidence interval; M-H: Mantel-Haenszel. Supplement Figure 2: forest plots of risk ratio in SRR at month 6. RTX: rituximab. CI: confidence interval; M-H: Mantel-Haenszel. Supplement Figure 3: forest plots of risk ratio in SAEs. RTX: rituximab. CI: confidence interval; M-H: Mantel-Haenszel. Supplement Table 1: baseline characteristics of included studies of the literature review. Supplement Table 2: subgroup and sensitivity analyses for the difference between the low-dose RTX plus combination therapy subgroup and low-dose RTX subgroup. Supplement Table 3: subgroup and sensitivity analyses for the difference between the standard-dose RTX plus combination therapy subgroup and the standard-dose RTX subgroup.
