# Supplementary material for: The Efficacy and Safety of Different Dosages of Rituximab for Adults with Immune Thrombocytopenia: A Systematic Review and Meta-Analysis
Source: Biomed Res Int. 2021 Oct 6;2021:9992086. doi: 10.1155/2021/9992086 (PMC8514896; doi:10.1155/2021/9992086)
Supplement: Supplementary Materials — Supplement Figure 1: forest plots of risk ratio in CRR after removing the research of Huang et al. RTX: rituximab. CI: confidence interval; M-H: Mantel-Haenszel. Supplement Figure 2: forest plots of risk ratio in SRR at month 6. RTX: rituximab. CI: confidence interval; M-H: Mantel-Haenszel. Supplement Figure 3: forest plots of risk ratio in SAEs. RTX: rituximab. CI: confidence interval; M-H: Mantel-Haenszel. Supplement Table 1: baseline characteristics of included studies of the literature review. Supplement Table 2: subgroup and sensitivity analyses for the difference between the low-dose RTX plus combination therapy subgroup and low-dose RTX subgroup. Supplement Table 3: subgroup and sensitivity analyses for the difference between the standard-dose RTX plus combination therapy subgroup and the standard-dose RTX subgroup. [file 9992086.f1.docx]

**SUPPLEMENTAL DATA**

**The efficacy and safety of different dosages of rituximab for adults with immune thrombocytopenia: a systematic review and meta-analysis**

Yu Dong^1^, Ming Yue^2^, Mengjiao Hu^2#^

**Supplementary File: Search strategy**

**Medline search strategy**

#1: Purpura, Thrombocytopenic, Idiopathic[mesh]

#2: immune thrombocytopenia

#3: ITP

#4: autoimmune thrombocytopenia

#5: Immune Thrombocytopenic Purpura

#6: Autoimmune Thrombocytopenic Purpura

#7: #1 or #2 or #3 or #4 or #5 or #6

#8: rituximab[mesh]

#9: rituxan

#10: GP2013

#11: anti-CD20

#12: IDEC-C2B8

#13: mabthera

#14: #8 or #9 or #10 or #11 or #12 or #13

#15: #7 and #14


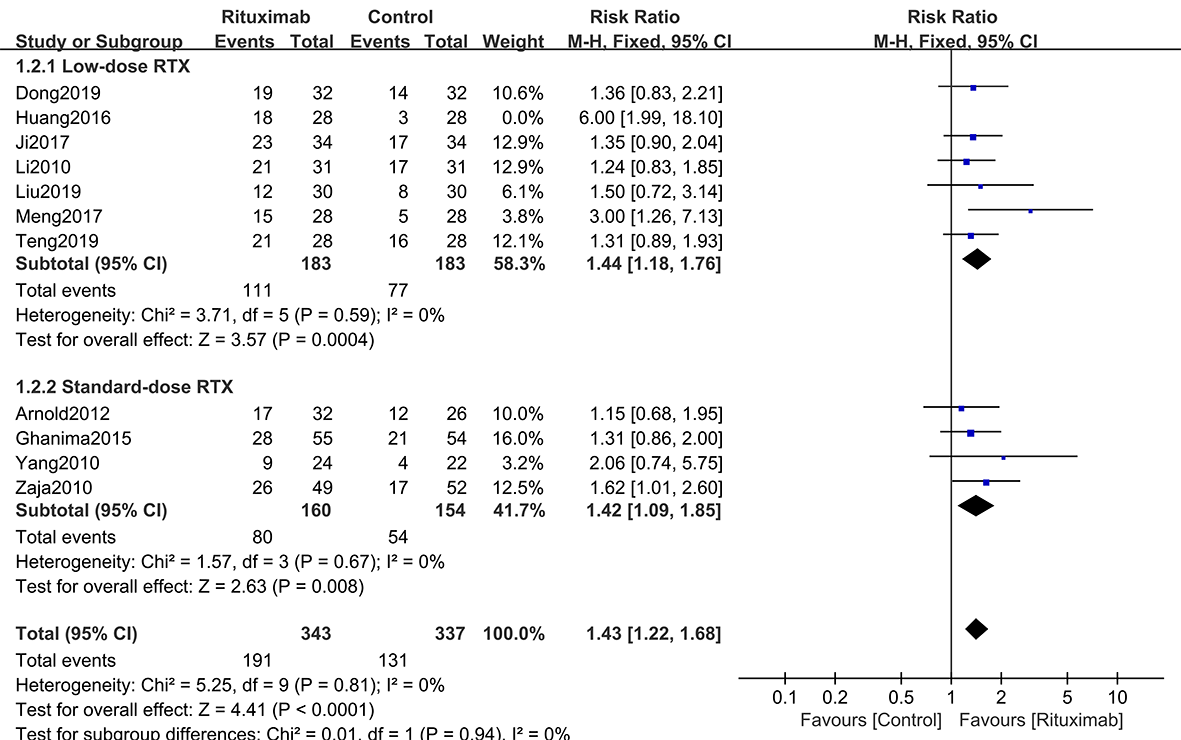


Supplement figure1. Forest plots of risk ratio in CRR after removing the research of Huang et al. RTX: rituximab. CI: confidence interval; M-H: Mantel-Haenszel.


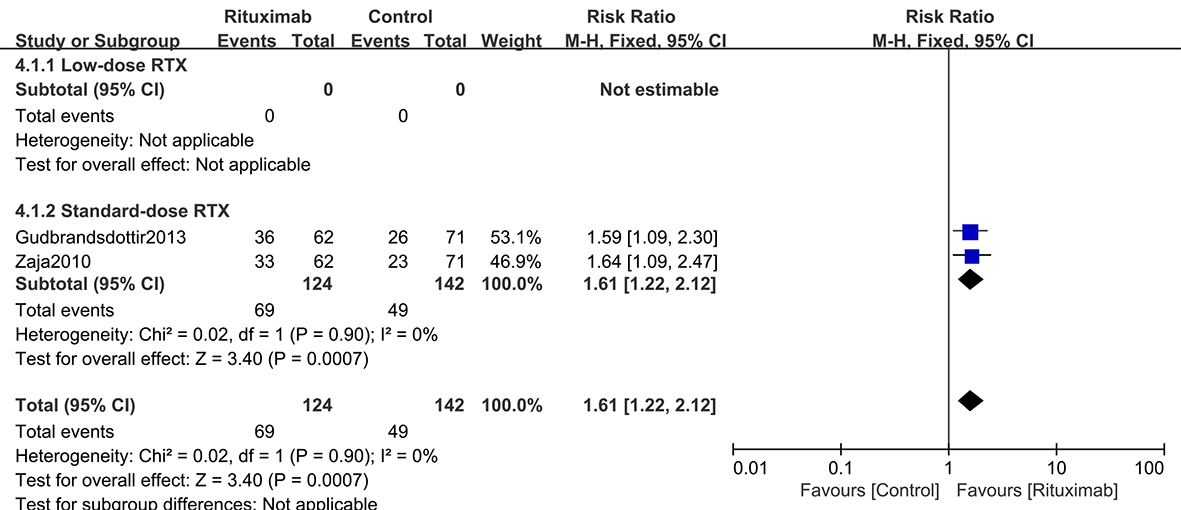


Supplement figure 2. Forest plots of risk ratio in SRR at month 6. RTX: rituximab. CI: confidence interval; M-H: Mantel-Haenszel.


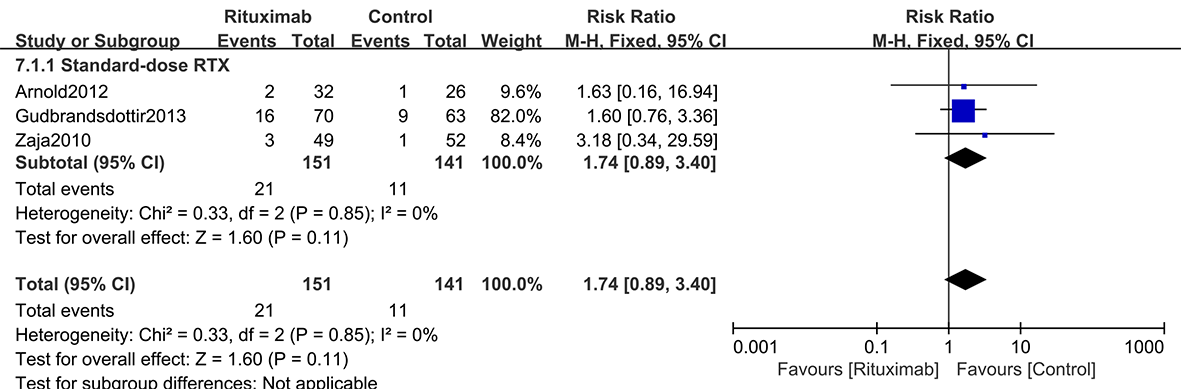


Supplement figure 3. Forest plots of risk ratio in SAEs. RTX: rituximab. CI: confidence interval; M-H: Mantel-Haenszel.

Supplement table 1. Baseline characteristics of included studies of the literature review.

|  |  |  | Response to treatment, N (%) | | | |  |
| --- | --- | --- | --- | --- | --- | --- | --- |
| Study | Patients, n | Intervention | Complete response,  >100 x 10^9^/L or  > 150 x 10^9^/L | Overall response,  >50 x 10^9^/L | Partial response,  >30 x 10^9^/L | | |
| **Low-dose RTX monotherapy** | | | | | |  |  |
| Alhassan et al. 2014 ^[1]^ | 13 | RTX: 100 mg, weekly, 28d | 4 (31) | 7 (54) | ND | | |
| Biondo et al. 2010 ^[2]^ | 10 | RTX: 100 mg, weekly, 28d | 4 (40) | 6 (60) | 7 (70) | | |
| Gracie et al. 2018 ^[3]^ | 179 | RTX: 100 mg, weekly, 28d | 48 (27) | ND | 102 (57) | | |
| Kapoor et al. 2017 ^[4]^ | 21 | RTX: 100 mg, weekly, 28d | 7 (33) | 10 (48) | ND | | |
| Kapoor et al. 2009 ^[5]^ | 10 | RTX: 100 mg, weekly, 28d | 4 (40) | 6 (60) | ND | | |
| Li et al. 2012 ^[6]^ | 20 | RTX: 100 mg, weekly, 28d | 11 (55) | 15 (75) | ND | | |
| Provan et al. 2007 ^[7]^ | 7 | RTX: 100 mg, weekly, 28d | 4 (57) | ND | 4 (57) | | |
| Raza et al. 2012 ^[8]^ | 6 | RTX: 100 mg, weekly, 28d | 0 (0) | 1 (17) | 2 (33) | | |
| Sui et al. 2010 ^[9]^ | 26 | RTX: 100 mg, weekly, 28d | 6 (23) | ND | 16 (62) | | |
| Sui et al. 2011 ^[10]^ | 31 | RTX: 100 mg, weekly, 28d | 9 (29) | ND | 18 (58) | | |
| Wang et al. 2019 ^[11]^ | 79 | RTX: 100 mg, weekly, 28d | 33 (42) | 58 (73) | Nd | | |
| Zaja et al. 2008 ^[12]^ | 28 | RTX: 100 mg, weekly, 28d | 12 (43) | 21 (75) | ND | | |
| Zaja et al. 2010 ^[13]^ | 48 | RTX: 100 mg, weekly, 28d | 19 (40) | 29 (60) | ND | | |
| Zaja et al. 2012 ^[14]^ | 25 | RTX: 100 mg, weekly, 28d | 7 (28) | ND | 13 (52) | | |
| **Low-dose RTX plus standard-of-care treatment** | | | | | |  |  |
| Zhou et al. 2019 ^[15]^ | 18 | RTX: 100 mg, weekly, 28d  DXM: 40 mg, qd, 1-4d | 12 (67) | ND | 18 (100) | | |
| Yan et al. 2015 ^[16]^ | 65 | RTX: 100 mg, weekly, 28d  DXM: 40 mg, qd, 1-4d | 47 (72) | ND | 53 (82) | | |
| Xing et al. 2013 ^[17]^ | 36 | RTX: 100 mg, weekly, 28d  DXM: 40 mg, qd, 1-4d | ND | 24 (67) | ND | | |
| Liu et al. 2019 ^[18]^ | 79 | RTX: 100 mg, weekly, 28d  DXM: 40 mg, qd, 1-4d | ND | 63 (80) | ND | | |
| Feng et al. 2015 ^[19]^ | 10 | RTX: 100 mg, weekly, 28d  DXM: 40 mg, qd, 1-4d | ND | ND | 10 (100) | | |
| Gomez-Almaguer et al. 2012 ^[20]^ | 21 | RTX: 100 mg, weekly, 28d  DXM: 40 mg, qd, 1-4d | 16 (76) | 19 (90) | ND | | |
| Dolai et al. 2016 ^[21]^ | 15 | RTX: 100 mg, weekly, 28d  DXM: 40 mg, qd, 1-4d and 15-18d | 9 (60) | ND | 11 (73) | | |
| Mukherjee et al. 2016 ^[22]^ | 22 | RTX: 100 mg, weekly, 28d  DXM: 40 mg, qd, 1-4d and 15-18d | 13 (59) | ND | 15 (68) | | |
| Xing et al. 2013 ^[17]^ | 38 | RTX: 100 mg, weekly, 28d  DXM: 40 mg, qd, 1-4d  PRE: taper, 28d | ND | 32 (84) | ND | | |
| Wang et al. 2019 ^[11]^ | 84 | RTX: 100 mg, weekly, 28d，  CTX: 2 mg/kg, qd, 2-3m | 58 (69) | 75 (89) | ND | | |

RTX: rituximab; DXM: dexamethasone; PRE: prednisone; CTX: cyclophosphamide; ND: no data; qd: once a day;

Supplement table 2. Subgroup and Sensitivity Analyses for the difference between low-dose RTX plus combination therapy subgroup and low-dose RTX subgroup.

| Low-dose RTX subgroup, or  low-dose RTX plus combination therapy subgroup | RR (risk ratio, 95% CI) | Test for overall effect  (P-Value) | Test for the difference between  low-dose RTX plus combination therapy subgroup  and low-dose RTX subgroup  (P-value) |
| --- | --- | --- | --- |
| **CRR (complete response rate)** |  | | |
| Low-dose RTX subgroup^[26, 28-33]^ | 1.61 [1.32, 1.97] | < 0.00001 | - |
| 100 mg RTX + 40 mg DXM *vs* 40 mg DXM^[28, 30，32]^ | 1.58 [1.17, 2.14] | 0.003 | 0.92 |
| 100 mg RTX + 40 mg DXM + PRE *vs* 40 mg DXM + PRE^[26]^ | 1.24 [0.83, 1.85] | 0.30 | 0.24 |
| 100 mg RTX + 3 mg tid DXM *vs* 3 mg tid DXM^[31]^ | 1.50 [0.72, 3.14] | 0.28 | 0.85 |
| 100 mg RTX + 1mg/kg DXM *vs* 1mg/kg DXM^[33]^ | 1.31 [0.89, 1.93] | 0.17 | 0.35 |
| 100 mg RTX + 2 mg/kg CTX *vs* 2 mg/kg CTX^[29]^ | 6.00 [1.99, 18.10] | 0.001 | 0.02 |
| **ORR (overall response rate)** |  | | |
| Low-dose RTX subgroup^[26, 29, 30]^ | 1.26 [1.06, 1.50] | 0.009 | - |
| 100 mg RTX + 40 mg DXM *vs* 40 mg DXM^[30]^ | 1.23 [0.91, 1.66] | 0.18 | 0.87 |
| 100 mg RTX + 40 mg DXM + PRE *vs* 40 mg DXM + PRE^[26]^ | 1.09 [0.83, 1.42] | 0.54 | 0.36 |
| 100 mg RTX + 2 mg/kg CTX *vs* 2 mg/kg CTX^[29]^ | 1.56 [1.11, 2.21] | 0.01 | 0.28 |
| **PRR (partial response rate)** |  | | |
| Low-dose RTX subgroup^[28, 33]^ | 1.25 [1.05, 1.48] | 0.01 | - |
| 100 mg RTX + 40 mg DXM *vs* 40 mg DXM^[28]^ | 1.25 [1.00, 1.56] | 0.05 | 1.00 |
| 100 mg RTX + 1 mg/kg DXM *vs* 1mg/kg DXM^[33]^ | 1.25 [0.96, 1.63] | 0.10 | 1.00 |
| **SRR (sustained response rate) at month 12** |  |  |  |
| Low-dose RTX subgroup^[26]^ | 2.00 [1.24, 3.24] | 0.005 | - |
| 100 mg RTX + 40 mg DXM + PRE *vs* 40 mg DXM + PRE^[26]^ | 2.00 [1.24, 3.24] | 0.005 | 1.00 |
| **Infection rate** |  | | |
| Low-dose RTX subgroup^[29, 31, 32]^ | 0.85 [0.28, 2.56] | 0.77 | - |
| 100 mg RTX + 40 mg DXM *vs* 40 mg DXM^[32]^ | 5.00 [0.25, 99.67] | 0.29 | 0.28 |
| 100 mg RTX + 3 mg tid DXM *vs* 3 mg tid DXM^[31]^ | 0.20 [0.02, 1.61] | 0.13 | 0.23 |
| 100 mg RTX + 2 mg/kg CTX *vs* 2 mg/kg CTX^[29]^ | 2.00 [0.19, 20.82] | 0.56 | 0.52 |
| **SB rate (significant bleeding rate)** |  | | |
| Low-dose RTX subgroup^[29]^ | 0.14 [0.01, 2.64] | 0.19 | - |
| 100 mg RTX + 2 mg/kg CTX vs 2 mg/kg CTX^[29]^ | 0.14 [0.01, 2.64] | 0.19 | 1.00 |

RTX: rituximab; DXM: dexamethasone; PRE: prednisone; CTX: cyclophosphamide; tid: three times a day; CRR: complete response rate (platelet count ≥ 100 × 10^9^/L); ORR: overall response rate (platelet count ≥50 × 10^9^/L); PRR: partial response rate (platelet count ≥ 30 × 10^9^/L); SB: significant bleeding.

Supplement table 3. Subgroup and Sensitivity Analyses for the difference between the standard-dose RTX plus combination therapy subgroup and the standard-dose RTX subgroup.

| Standard-dose RTX subgroup, or  standard -dose RTX plus combination therapy subgroup | RR (risk ratio, 95% CI) | Test for overall effect  (P-Value) | Test for the difference between the  standard-dose RTX plus combination therapy subgroup  and the standard-dose RTX subgroup  (P-value) |
| --- | --- | --- | --- |
| **CRR (complete response rate)** |  | | |
| Standard-dose RTX subgroup^[23, 24, 27, 34]^ | 1.42 [1.09, 1.85] | 0.008 | - |
| 375 mg/m^2^ RTX + 40 mg DXM *vs* 40 mg DXM^[27, 34]^ | 1.71 [1.11, 2.63] | 0.01 | 0.47 |
| 375 mg/m^2^ RTX *vs* Placebo^[23, 24]^ | 1.25 [0.90, 1.74] | 0.19 | 0.54 |
| **ORR (overall response rate)** |  | | |
| Standard-dose RTX subgroup^[25, 27, 34]^ | 1.49 [1.16, 1.91] | 0.002 | - |
| 375 mg/m^2^ RTX + 40 mg DXM *vs* 40 mg DXM^[25, 27, 34]^ | 1.49 [1.16, 1.91] | 0.002 | 1.00 |
| **PRR (partial response rate)** |  | | |
| Standard-dose RTX subgroup^[23, 24]^ | 1.00 [0.82, 1.23] | 0.96 | - |
| 375 mg/m^2^ RTX *vs* Placebo^[23, 24]^ | 1.00 [0.82, 1.23] | 0.96 | 1.00 |
| **SRR (sustained response rate) at month 6** |  | | |
| Standard-dose RTX subgroup^[25, 27]^ | 1.61 [1.22, 2.12] | 0.0007 | - |
| 375 mg/m^2^ RTX + 40 mg DXM *vs* 40 mg DXM^[25, 27]^ | 1.61 [1.22, 2.12] | 0.0007 | 1.00 |
| **SRR (sustained response rate) at month 12** |  | | |
| Standard-dose RTX subgroup^[25]^ | 1.64 [1.09, 2.47] | 0.02 | - |
| 375 mg/m^2^ RTX + 40 mg DXM *vs* 40 mg DXM^[25]^ | 1.64 [1.09, 2.47] | 0.02 | 1.00 |
| **Infection rate** |  | | |
| Standard-dose RTX subgroup^[23, 24, 25, 27]^ | 1.46 [0.97, 2.20] | 0.07 | - |
| 375 mg/m^2^ RTX + 40 mg DXM *vs* 40 mg DXM^[25, 27]^ | 1.73 [0.80, 3.72] | 0.16 | 0.70 |
| 375 mg/m^2^ RTX *vs* Placebo^[23, 24]^ | 1.35 [0.83, 2.17] | 0.22 | 0.80 |
| **SB rate (significant bleeding rate)** |  | | |
| Standard-dose RTX subgroup^[23, 24, 25, 27]^ | 1.19 [0.62, 2.27] | 0.61 | - |
| 375 mg/m^2^ RTX + 40 mg DXM *vs* 40 mg DXM^[25, 27]^ | 3.32 [0.53, 20.88] | 0.20 | 0.30 |
| 375 mg/m^2^ RTX *vs* Placebo^[23, 24]^ | 0.93 [0.46, 1.90] | 0.85 | 0.63 |
| **SAEs (severe adverse events)** |  | | |
| Standard-dose RTX subgroup^[23, 24, 27]^ | 1.74 [0.89, 3.40] | 0.11 |  |
| 375 mg/m^2^ RTX + 40 mg DXM *vs* 40 mg DXM^[27]^ | 1.75 [0.87, 3.53] | 0.12 | 0.99 |
| 375 mg/m^2^ RTX *vs* Placebo^[23, 24]^ | 1.63 [0.16, 16.94] | 0.68 | 0.96 |

RTX: rituximab; DXM: dexamethasone; PRE: prednisone; CTX: cyclophosphamide; tid: three times a day; CRR: complete response rate (platelet count ≥ 100 × 10^9^/L); ORR: overall response rate (platelet count ≥ 50 × 10^9^/L); PRR: partial response rate (platelet count ≥ 30 × 10^9^/L); SB: significant bleeding.

**References**

1. Alhassan S, Bennett SA, Davies K, Evans G. Use of single agent low dose rituximab in primary immune thrombocytopenia and warm autoimmune haemolytic anaemia. British Journal of Haematology. 2014;165:28.

2. Biondo F, Santoro C, Baldacci E, Cafolla A, De Propris MS, Guarini A, et al. Low-dose rituximab in adult patients with persistent/chronic primary immune thrombocytopenia: A single center experience. Haematologica. 2010;95:81.

3. Gracie C, Zaidi A, Doobaree U, Taylor L, Provan D, Newland A, et al. Comparison of standard-and low-dose rituximab in primary immune thrombocytopenia (ITP): Data from the UK ITP registry. HemaSphere. 2018;2:23.

4. Kapoor R, Kumar R, Mahapatra M, Pati HP, Pramanik SK. Low Dose Rituximab in Chronic ITP: Still an Option in Resource Limited Settings. Indian J Hematol Blood Transfus. 2017;33:568-73.

5. Kapoor R, Mahapatra M, Pati HP, Mishra P. To study the efficacy and safety of low dose rituximab in chronic immune thrombocytopenic purpura (ITP). Indian Journal of Hematology and Blood Transfusion. 2009;25:141-2.

6. Li Y, Wang XM, Mao M, Zhang XY, Fu L, Ai HM, et al. [Clinical efficacy of lower dose rituximab for chronic refractory immune thrombocytopenic purpura]. Zhonghua Xue Ye Xue Za Zhi. 2012;33:204-6.

7. Provan D, Butler T, Evangelista ML, Amadori S, Newland AC, Stasi R. Activity and safety profile of low-dose rituximab for the treatment of autoimmune cytopenias in adults. Haematologica. 2007;92:1695-8.

8. Raza K, Murphy P, Quinn J, Thornton P. An experience with low dose rituximab for the treatment of patients with chronic immune thrombocytopenic purpura: A retrospective analysis of six patients. Haematologica. 2012;97:683.

9. Sui T, Xue F, Zhao HF, Ge J, Zhou H, Zhang L, et al. [Efficacy of lower dose rituximab therapy for idiopathic thrombocytopenic purpura.]. Zhonghua Xue Ye Xue Za Zhi. 2010;31:161-3.

10. Sui T, Zhang L, Zhou ZP, Xue F, Ge J, Yang RC. [Efficacy and safety of two different low-dose rituximab regimens for Chinese adult patients with immune thrombocytopenia]. Zhonghua Xue Ye Xue Za Zhi. 2011;32:583-6.

11. Wang J, Wang B, Sun Z, Xue K. Therapeutic effects of rituximab combined with cyclophosphamide on refractory idiopathic thrombocytopenic purpura. Exp Ther Med. 2019;17:2137-42.

12. Zaja F, Battista ML, Pirrotta MT, Palmieri S, Montagna M, Vianelli N, et al. Lower dose rituximab is active in adults patients with idiopathic thrombocytopenic purpura. Haematologica. 2008;93:930-3.

13. Zaja F, Vianelli N, Volpetti S, Battista ML, Defina M, Palmieri S, et al. Low-dose rituximab in adult patients with primary immune thrombocytopenia. Eur J Haematol. 2010;85:329-34.

14. Zaja F, Volpetti S, Chiozzotto M, Puglisi S, Isola M, Buttignol S, et al. Long-term follow-up analysis after rituximab salvage therapy in adult patients with immune thrombocytopenia. Am J Hematol. 2012;87:886-9.

15. Zhou H, Liu L, Shu X, Wang X, Song Y. Clinical Efficacy and Safety of High-Dose Dexamethasone Plus Low-Dose Rituximab as First-Line Therapy in Newly Diagnosed Primary Immune Thrombocytopenia. Indian J Hematol Blood Transfus. 2019;35:507-12.

16. Yan Z, Li Z, Zhang H, Chen C, Li D, Xing W, et al. [Efficacy of high-dose dexamethasone plus low-dose rituximab as a second-line treatment in 65 patients with primary immune thrombocytopenia]. Zhonghua Xue Ye Xue Za Zhi. 2015;36:206-9.

17. Xing WW, Li ZY, Yan ZL, Liu KG, Li N, Cao J, et al. [Efficacy and safety of low-dose rituximab combined with different dosage of glucocorticoids for immune thrombocytopenia]. Zhonghua Xue Ye Xue Za Zhi. 2013;34:409-12.

18. Liu JX, Zhang HX, Li DP, Xing WW, Li HJ, Chen W, et al. [Curative Efficacy of Rituximab for ITP Patients with Different Sensitivity to Hormone]. Zhongguo Shi Yan Xue Ye Xue Za Zhi. 2019;27:1602-6.

19. Feng K. Clinical observation of 10 cases of newly diagnosed itp patients receiving low-dose rituximab combined with high-dose dexamethasone. Journal of Thrombosis and Haemostasis. 2015;13:870.

20. Gomez-Almaguer D, Tarin-Arzaga LC, Moreno-Jaime B, Jaime-Pérez JC, Ceballos-López AA, Ruiz-Argüelles GJ, et al. Low-dose rituximab and high-dose dexamethasone as front-line therapy in adult patients with primary immune thrombocytopenia. Blood. 2012;120.

21. Dolai TK, Mukherjee S, Mandal PK, De R, Chakrabarti P. A prospective study to evaluate the efficacy, safety and response duration of newly diagnosed adult immune thrombocytopenia patients treated with low dose rituximab and high dose of dexamethasone. Blood. 2016;128.

22. Mukherjee S, Dolai TK, Mandal PK, De R, Chakrabarti P. A Prospective study to evaluate the efficacy, safety and response duration of newly diagnosed Adult ITP patients treated with low dose rituximab and high dose of dexamethasone. Indian Journal of Hematology and Blood Transfusion. 2016;32:S383.

23. Arnold DM, Heddle NM, Carruthers J, Cook DJ, Crowther MA, Meyer RM, et al. A pilot randomized trial of adjuvant rituximab or placebo for nonsplenectomized patients with immune thrombocytopenia. Blood. 2012;119:1356-62.

24. Ghanima W, Khelif A, Waage A, Michel M, Tjønnfjord GE, Romdhan NB, et al. Rituximab as second-line treatment for adult immune thrombocytopenia (the RITP trial): a multicentre, randomised, double-blind, placebo-controlled trial. The Lancet. 2015;385:1653-61.

25. Gudbrandsdottir S, Birgens HS, Frederiksen H, Jensen BA, Jensen MK, Kjeldsen L, et al. Rituximab and dexamethasone vs dexamethasone monotherapy in newly diagnosed patients with primary immune thrombocytopenia. Blood. 2013;121:1976-81.

26. Li Z, Mou W, Lu G, Cao J, He X, Pan X, et al. Low-dose rituximab combined with short-term glucocorticoids up-regulates Treg cell levels in patients with immune thrombocytopenia. Int J Hematol. 2011;93:91-8.

27. Zaja F, Baccarani M, Mazza P, Bocchia M, Gugliotta L, Zaccaria A, et al. Dexamethasone plus rituximab yields higher sustained response rates than dexamethasone monotherapy in adults with primary immune thrombocytopenia. Blood. 2010;115:2755-62.

28. Dong S, Zhao Y. Clinical effect of low-dose rituximab in the treatment of refractory thrombocytopenic purpura. Systems Medicine. 2019;4:1-3,59. https://kns.cnki.net/kcms/detail/detail.aspx?dbcode=CJFD&dbname=CJFDLAST2019&filename=XTYX201915001

29. Huang Y, Liu Y, Wang W. Clinical observation of rituximab combined with cyclophosphamide treatment on refractory idiopathic thrombocytopenic purpura. Medical Recapitulate. 2016;22:572-5. <https://kns.cnki.net/kcms/detail/detail.aspx?dbcode=CJFD&dbname=CJFDLAST2016&filename=YXZS201603048>

30. Ji C, Li H. Efficacy and safety of low-dose rituximab combined with high-dose dexamethasone in the treatment of immune thrombocytopenia. World Latest Medicine Information (Electronic Version). 2017;17:117-8. https://kns.cnki.net/kcms/detail/detail.aspx?dbcode=CJFD&dbname=CJFDLAST2017&filename=WMIA201702104

31. Liu C, Luan L. Effect of glucocorticoid combined with low-dose rituximab injection in the treatment of refractory primary immune thrombocytopenia. China Modern Medicine. 2019;26:68-70,4. https://kns.cnki.net/kcms/detail/detail.aspx?dbcode=CJFD&dbname=CJFDLAST2019&filename=ZGUD201930019

32. Meng J, Long C. Clinical study of glucocorticoids combined with low-dose of rituximab in the treatment of refractory primary immune thrombocytopenia. Clinical Medicine. 2017;37:21-2. https://kns.cnki.net/kcms/detail/detail.aspx?dbcode=CJFD&dbname=CJFDLAST2017&filename=EBED201702010

33. Teng Z, Lei Q, Xu S, Wang J. Clinical study of rituximab in patients with refractory idiopathic thrombocytopenic purpura. Shanxi Medical Journal. 2019;48:102-4. https://kns.cnki.net/kcms/detail/detail.aspx?dbcode=CJFD&dbname=CJFDLAST2019&filename=SXYZ201901032

34. Yang H. Observation of the clinical effect of mabthera combined with dexamethasone in the treatment of refractory idiopathic thrombocytopenic purpura. Aerospace Medicine. 2010;21:2024-5. https://kns.cnki.net/kcms/detail/detail.aspx?dbcode=CJFD&dbname=CJFD2010&filename=HKHT201011052
